# Microorganisms with Claimed Probiotic Properties: An Overview of Recent Literature

**DOI:** 10.3390/ijerph110504745

**Published:** 2014-05-05

**Authors:** Sabina Fijan

**Affiliations:** Faculty of Health Sciences, University of Maribor, Žitna ulica 15, 2000 Maribor, Slovenia; E-Mail: sabina.fijan@um.si; Tel.: +386-2-300-47-52

**Keywords:** Lactic-acid bacteria, *Lactobacillus*, *Bifidobacterium*, *Saccharomyces*, *Enterococcus*, *Streptococcus*, *Pediococcus*, *Leuconostoc*, *Bacillus*, *Escherichia coli*

## Abstract

Probiotics are defined as live microorganisms, which when administered in adequate amounts, confer a health benefit on the host. Health benefits have mainly been demonstrated for specific probiotic strains of the following genera: *Lactobacillus*, *Bifidobacterium*, *Saccharomyces*, *Enterococcus*, *Streptococcus*, *Pediococcus, Leuconostoc*, *Bacillus*, *Escherichia coli*. The human microbiota is getting a lot of attention today and research has already demonstrated that alteration of this microbiota may have far-reaching consequences. One of the possible routes for correcting dysbiosis is by consuming probiotics. The credibility of specific health claims of probiotics and their safety must be established through science-based clinical studies. This overview summarizes the most commonly used probiotic microorganisms and their demonstrated health claims. As probiotic properties have been shown to be strain specific, accurate identification of particular strains is also very important. On the other hand, it is also demonstrated that the use of various probiotics for immunocompromised patients or patients with a leaky gut has also yielded infections, sepsis, fungemia, bacteraemia. Although the vast majority of probiotics that are used today are generally regarded as safe and beneficial for healthy individuals, caution in selecting and monitoring of probiotics for patients is needed and complete consideration of risk-benefit ratio before prescribing is recommended.

## 1. Background

It is scientifically established that certain species of microorganisms make us sick and can even kill us. Among the deadliest microorganisms throughout history, either due to global number of deaths, mortality rate or production of lethal toxins, we usually include *Yersinia pestis*, influenza virus, AIDS/HIV virus, *Clostridium tetani*, *Mycobacterium tuberculosis* and *Vibrio cholerae*, just to name a few. Recently; many multi-drug resistant bacteria have been causing important health-care associated infections and dangerous serotypes have been causing serious emerging food-poisonings due to production of enterotoxins. Some of these medically important bacteria include: methicillin-resistant *Staphylococcus aureus* (MRSA), vancomycin-resistant enterococci (VRE), extended-spectrum beta-lactamase (ESBL) producing Enterobacteriaceae, multi-drug resistant *Pseudomonas aeruginosa*, multi-drug resistant *Mycobacterium tuberculosis* and Enterohemorrhagic *Escherichia coli* (EHEC) [1,2].

Therefore; throughout the history of microbiology, most human studies have been focused on the disease-causing organisms found on or in people; whilst fewer studies have examined the benefits of the resident bacteria [3]. As noted in several reviews [4,5] the endogenous flora of the human body is poorly understood. However, we are surrounded by an important biological system of microorganisms that live in or on the human body and are beneficial. This biological system is the human microbiome. It has been known for some time that the human body is inhabited by at least 10 times more bacteria than the number of human cells in the body, and that the majority of those bacteria are found in the human gastrointestinal tract [3,6]. The composition of the gut microbiota varies during childhood until the individual reaches adulthood [7]. The relationship between the host and the gut microbiota is symbiotic. The mainly commensal intestinal microbiota contribute to the enhancing resistance against infections, differentiation of the host immune system, synthesis of certain nutrients such as vitamins, short-chain fatty acids and other low molecular mass molecules. However, little is really systematically known about the body of evidence to evaluate the role of the gut indigenous microflora and the consequences of microecological imbalances as well as the metabolic consequences that may impact progression of metabolic disease [8,9].

After the successful human genome project that mapped the entire human genome, the International Consortium on the Microbiome has launched similar projects of mapping the human microbiome with two major arms: the NIH Human microbiome project and the EU FP 7 MetaHit Project [3]. An important observation is that, while our health is certainly influenced by genes, it may also be even more powerfully influenced by our microbiome [10]. Although human microbiota studies have already been conducted, projects that investigate patient perceptions of bioengineered probiotics and analysing existing regulatory frameworks for the federal regulation of probiotics are of interest to public health and are therefore funded.

## 2. Probiotics as Microorganisms with Health Benefits

According to the Food and Agriculture Organisation of the United Nations (FAO) and the World Health Organisation (WHO) [11,12] probiotics are defined as live microorganisms, which when administered in adequate amounts, confer a health benefit on the host. This definition of probiotics is also adopted by the International Scientific Association for Probiotics and Prebiotics (ISAPP) and is used in most scientific publications. However, this definition is not accepted by European Food Safety Authority (EFSA) or the U.S. Food and Drug Administration (FDA) [13,14] at the moment since they insist that the health claim incorporated in the definition is not measurable due to the fact that commercial markets have outpaced the ability of science to substantiate the evidence. As a consequence, no substantiated health claims have been approved for any probiotic in the U.S. [15]. However, health claims, such as treatment and cures of disease using probiotics, are measurable and can be proven with similar type of studies as are conducted for drugs (double-blinded, randomized, placebo-controlled human trials). Although the benefits of consuming fermented foods have been known to humankind for centuries; long before microorganisms were discovered; the concept of administering microorganisms in order to confer a positive health benefit started over a century ago when Metchnikoff theorized that health could be enhanced, and also senility could be delayed, by manipulating the intestinal microbiome with host-friendly bacteria found in yogurt [16].

Over the last two decades there has been growing interest on both basic and clinical science in probiotics which has resulted in over 6000 publications in the biomedical literature, with over 60% published in the last 5 years, some in the top ranking scientific journals [14]. The most common types of microorganisms used as probiotics are lactic acid bacteria and bifidobacteria, although other bacteria and certain yeasts are also used [17]. It is important to stress that the biological effects of probiotics are strain specific and that the success or failure of one strain cannot be extrapolated to another strain. Thus proper strain identification using novel molecular and based technologies is imperative [18]. Species identification can be performed by 16S rRNA gene sequence analysis and DNA-DNA hybridisation techniques. Strain identification can further be performed by various reproducible molecular methods or using unique phenotypic traits. Examples of molecular methods are: pulsed field gel electrophoresis and randomly amplified polymorphic DNA. Phenotype traits for strain identification include: determining the presence of extrachromosomal genetic elements, fermentation of a range of sugars and detection of final fermentation products obtained from glucose utilization [12].

Another important aspect to mention when claiming the health benefits of probiotics is that a beneficial effect can only be demonstrated by *in vivo* studies [12]. Although *in vitro* studies or animal models cannot prove a probiotic effect, they can be used to characterise a possible mechanism of probiotic action, determine the safety of probiotic microorganisms or convey other knowledge of probiotic strains. Thus, *in vitro* studies provide the first step in evaluating probiotics for food use and should be followed by double-blinded, randomized, placebo-controlled human trials. Appropriate target-specific *in vitro* studies that correlate with *in vivo* are recommended. For example *in vitro* bile salts resistance was shown to correlate with gastric survival *in vivo* [19]. Knowledge on probiotic strains is therefore the first step conducted through *in vitro* studies. The most important properties of strains to even be considered for probiotic use include: resistance to gastric acidity, bile acid resistance, adherence to mucus and/or human epithelial cells and cell lines, antimicrobial activity against potentially pathogenic bacteria or fungi, ability to reduce pathogen adhesion to surfaces, bile salt hydrolase activity, enhancing viability of probiotics. [12,20,21,22,23,24].

The aim of this overview of recent literature was to present the most common microorganisms with their recently claimed probiotic properties. 

## 3. Methods

A literature overview of the PubMed database using the keywords: ‘probiotic’ and ‘microorganisms’ yielded 920 publications. Of these; 682 were published in the last 10 years (74 %); and 492 were published in the last 5 years (53 %). Thomson Reuters Web of science yielded 1078 publications using the keywords: ‘probiotic’ and ‘microorganisms’ since 1970, of these 886 (82.1 %) were published in the last 10 years and 676 (62.7 %) were published in the last five years. The literature overview for this article included other databases such as ScienceDirect and Google search as well as other keyword combinations (individual microorganism/probiotic). Unless otherwise relevant, only a selection of the most recent articles focused on probiotic microorganisms and their health claims were chosen to be included in this overview. Of course, this does not mean that all important research on probiotic microorganisms was included.

## 4. Results and Discussion

### 4.1. Specific Strains with Probiotic Properties in the Genus Lactobacillus

The genus *Lactobacillus* includes various Gram positive facultative anaerobic or microaerophilic rod-shaped bacteria. They are a major part of the lactic acid bacteria (LAB) group (including *Lactobacillus*, *Lactococcus*, *Enterococcus*, *Oenococcus*, *Pediococcus*, *Streptococcus* and *Leuconostoc* species) that can convert hexose sugars to lactic acid thus producing an acid environment which inhibits the growth of several species of harmful bacteria [25]. In humans, *Lactobacilli* are normally present in the vagina, gastrointestinal tract [26] and are together with *Bifidobacterium* one of the first bacteria to colonize the infant gut after delivery [27]. The complete genome sequences of the following commensal *Lactobacilli* have been published: *Lactobacillus plantarum*, *L. johnsonii*, *L. acidophilus*, *L. sakei*, *L. bulgaricus*, *L. salivarius* [25].

Some *Lactobacilli* are used for the production of yogurt, cheese, sauerkraut, pickles, sourdough, wine and other fermented products [28,29,30]. In all cases, sugars are metabolised into lactic acid; thus creating a hostile environment for spoilage microorganisms and enabling food preservation.

It has been found that infants with food allergies exhibit an imbalance between beneficial and potentially harmful bacteria, *i.e.*, decreased *Lactobacillus*, *Bifidobacteria* and *Enterococcus* species and increased coliforms, *Staphylococcus aureus* and *Clostridium* species; suggesting that microbial inhabitants of the human body, may play either a pathogenic or protective role in allergies. Based on this data, many clinical trials addressing the use of probiotics in the context of allergic disorders have been conducted in children. However, currently, no conclusive item may be drawn [31].

*Lactobacilli* have received tremendous attention due to their health-promoting properties [26]. However, a very important fact that is sometimes overlooked by scientists is that most *Lactobacilli* do not form stable and numerically significant populations in the human intestinal tract, especially in the small intestine where they are presumed to form epithelial associations [26]. Similarly, in the research on modulating intestinal bifidobacteria and *Lactobacilli* after one week supplementation with commercial probiotic food supplements, only a significant increase in the intestinal *Lactobacillus acidophilus* group was observed and even this increase disappeared after a few days [32]. The authors concluded that short-term daily intake of live probiotic cells is insufficient in modulating the intestinal bifidobacteria and *Lactobacilli*. *Lactobacilli* such as: *Lactobacillus acidophilus*, *L. casei*, *L. paracasei*, *L. rhamnosus*, *L. delbrueckii* subsp. *bulgaricus*, *L. brevis*, *L. johnsonii*, *L. plantarum* and *L. fermentum* are commonly used as probiotic products. Although *Lactobacilli* are often described as indigenous inhabitants of the human intestinal tract, they are more likely to be autochthonous of the oral cavity or fermented foods [26,32].

Studies have shown that certain strains of *Lactobacilli* are effective in preventing antibiotic-associated diarrhoea [33,34]. *Lactobacilli* species are commonly selected as probiotics since they express many crucial properties such as: high tolerance to acid and bile, capability to adhere to intestinal surfaces, withstanding low pH, gastric juice, inhibiting potentially pathogenic species (antimicrobial activity), resisting antibiotics, producing exopolysaccharides and removing cholesterol [20,35,36]. *Lactobacillus rhamnosus* CRL1505 has even been effective in reducing viral-associated pulmonary damage through controlling immune-coagulative responses and clearing respiratory viruses [37]. In a published meta-analysis [38] it has been shown that even though the probiotic strains of *Lactobacillus* were safe and effective in preventing recurrent urinary tract infections in adult women, the authors concluded that more randomized clinical trials should be conducted to make a more definitive recommendation.

Sometimes probiotic ‘cocktails’ comprising of various strains are used [39,40,41]. Several probiotic strains of microorganisms are effective in competing against common causes of travellers’ diarrhoea [39,42] caused mostly by bacterial pathogens such as: one of the seven types of diarrheagenic *Escherichia coli*, *Campylobacter jejuni* and *Shigella* species. The most commonly used probiotic microorganisms against these pathogens are: *Lactobacillus acidophilus*, *Lactobacillus rhamnosus* GG, *Saccharomyces boulardii*, *Bifidobacterium bifidum* and *Bacillus coagulans*. Using *Lactobacilli* and other probiotics together with antibiotics also seems to be promising for the treatment of bacterial vaginosis, although more well-designed randomized controlled trials with standard methodologies and larger patient size are needed to inconclusively prove benefits [39].

The review of randomized control trials on the use of probiotics for functional constipation [43] revealed that the favourable treatment for adults was with *Bifidobacterium lactis* DN-173 010, *Lactobacillus casei* Shirota and *Escherichia coli* Nissle 1917. A beneficial effect on children was shown with *Lactobacillus casei rhamnosus* Lcr35. In the review of probiotic safety during pregnancy [41] it was found that *Lactobacillus* and *Bifidobacterium* had no effect on the incidence of Caesarean section, birth weight or gestational age and no malformations were reported. The study [44] monitoring one month administration of a multi-strain probiotic capsule containing: *Lactobacillus acidophilus*, *Lactobacillus delbrueckii* subsp. *bulgaricus*, *Bifidobacterium animalis* subsp. *lactis* and *Streptococcus thermophilus* gave indecisive results, as no difference in the colonic microflora of patients with colitis (Crohn’s colitis or ulcerative colitis) was observed.

On the other hand, a liver abscess caused by a *Lactobacillus rhamnosus* that was not distinguishable from the probiotic strain *L. rhamnosus* GG [45] and *Lactobacillus* endocarditis caused by *L. rhamnosus* (strain not specified) have been reported [46]. Although both reports [45,46] gave no information on the origin of infection; both reports highlight the importance of strain identification. It is also important to add that both articles were dated from the year 1999 when methods for exact strain identification were not as perfected as today.

### 4.2. Specific Strains with Probiotic Properties in the Genus Bifidobacterium

The genus *Bifidobacterium* includes various Gram positive non-motile anaerobic bacteria. They are endosymbiotic inhabitants of the gastrointestinal tract and vagina of mammals, including humans [47]. Strains of the genus *Bifidobacterium* are also often used as probiotic bacteria as they are known for their variety of resistance mechanisms to bile salts, which is important since the beneficial effects of probiotic bacteria must be generated in the presence of this biological fluid.

It has even been proven that although bile tolerance is strain dependent, both wild type-bile sensitive bifidobacteria and *Lactobacilli* strains can progressively adapt to the presence of bile salts by subculturing and gradually increasing concentration of bile [36,48]. Several strains of bifidobacteria are considered as important probiotics including: *Bifidobacterium infantis*, *B. adolescentis*, *B. animalis* subsp *animalis*, *B. animalis* subsp *lactis*, *B. bifidum*, *B. longum*, *B. breve*. Companies often use trademark names for some of these bifidobacteria by inventing scientific sounding commercial names.

As mentioned before *Bifidobacterium* species together with other probiotics have been proven to treat constipation [43], travellers’ diarrhoea [39], antibiotic-associated diarrhoea [34], maintaining remission of disease activity of gut inflammation and moderate ulcerative colitis [49,50], prevention as well as treatment of necrotizing enterocolitis in newborns [51], reduction of radiation induced diarrhoea [52], reducing the development of disease risk for eczema, food allergies [53], cholesterol-lowering capacities [36].

### 4.3. Specific Strains with Probiotic Properties in the Genus Saccharomyces

The genus *Saccharomyces* includes various yeasts such as: *Saccharomyces cerevisiae* (used for making wine, bread, beer), *Saccharomyces bayanus* (used for making wine) and *Saccharomyces boulardii* used in medicine as a probiotic. *Saccharomyces* yeasts also form symbiotic matrices with bacteria to form kefir [54] and are sometimes a component of kombucha [55].

*S. boulardii* is often marketed as a probiotic in a lyophilized form to treat diarrhoea while maintaining an excellent reputation for safety [39]. Most reports show a clinical benefit of *S. boulardii* in decreasing the duration of diarrhoea regardless of the cause and thus reducing hospital stay resulting in social and economic benefits [7,33,56,57,58]. Administration of *S. boulardii* has shown positive effects for patients with irritable bowel syndrome [59], preventing and treating relapses of inflammatory bowel disease and for treating moderate symptoms of ulcerative colitis [60,61]. Recurrent pseudomembranous colitis infection caused by *Clostridium difficile* can also be significantly reduced by administration of daily dosages of *S. boulardii* together with standard antibiotics [62]. In the previously mentioned review of probiotic safety during pregnancy [41] no malformations were reported. On the other hand, it must not be neglected that in immunocompromised individuals or other patients, *S. boulardii* can cause fungemia or localised infections [63,64].

### 4.4. Specific Strains with Probiotic Properties in the Genus Lactococcus

*Lactococcus* is a genus of Gram positive lactic acid bacteria that are commonly used in the dairy industry for manufacturing fermented products. They are important in preventing growth of spoilage bacteria in milk products due to acidification. They are also sometimes recommended as probiotics. Certain strains of *Lactococcus lactis* subsp. *lactis* have probiotic properties such as adhesion to vaginal epithelial cells and nisin production (*Lactococcus lactis* subsp. *lactis* CV56) [65,66] and are also used to treat antibiotic-associated diarrhoea in combination with other probiotics [33].

### 4.5. Specific Strains with Probiotic Properties in the Genera Streptococcus and Enterococcus

The genera *Streptococcus* and *Enterococcus* are also part of the lactic acid bacteria and contain several strains associated with severe health-care associated infections such as: *Streptococcus pyogenes*, *Streptococcus pneumoniae*, vancomycin-resistant *Enterococcus faecium* [67]. However, other strains form part of the commensal human microbiome of the mouth, skin, and intestine, such as *Enterococcus faecium* PC4.1 [68] and others. Some strains have probiotic properties: such as *Enterococcus durans* [69] and *Streptococcus thermophilus* [70] (also used for the production of yogurt alongside *Lactobacillus delbrueckii* subsp. *bulgaricus*).

Although *Enterococcus faecium* has a long history of probiotic use, especially in preventing antibiotic-associated diarrhoea [34], certain strains are opportunistic pathogens that present a potential reservoir of antibiotic resistance and virulence genes (animal study) [71] and are therefore generally not treated as safe (GRAS) for humans, but represent important probiotics for animals [72,73].

### 4.6. Specific Strains with Probiotic Properties in the Genus Bacillus

The genus *Bacillus* includes Gram positive spore-forming aerobic or facultative aerobic members with claimed probiotic properties including: *B. subtilis*, *B. coagulans*, *B. subtilis*, *B. cereus*.

*Bacillus coagulans* together with other microorganisms has proven to be most successful in preventing or treating antibiotic-associated diarrhoea [34,42].

*Bacillus subtilis* spores have been considered as probiotics for animal consumption [74,75] and have been proposed for treating diarrhoea and *H. pylori* eradication in humans [76]. However, a report of a recurrent septicaemia in an immunocompromised patient due to treatment with spores of the probiotic strain *B. subtilis* [77] and four cases of nosocomial bacteraemia caused by absorption of an oral preparation containing *B. subtilis* spores [78] have shown the high risk of using *Bacillus subtilis* spores as probiotics to immunocompromised patients, and that ingestion is safe only for humans under normal host conditions [79].

*B. cereus* NVH 75/95 has also proven to be an efficient probiotic for animals [80]. Certain *B. cereus* strains are human-lethal and highly toxic, whereas other strains have probiotic properties [81].

Another report [82] highlighted the importance of confirming strain identities thus avoiding casual links between probiotic microorganisms and strains isolated from immune-suppressed hosts. This report also proved that the cholangitis due to *Bacillus* in a French hospital was not caused by a probiotic.

### 4.7. Specific Strains with Probiotic Properties in the Genus Escherichia

Although the genus *Escherichia,* which belongs to the Gram negative family Enterobacteriaceae, is mainly known for its severely virulent serotypes (e.g., *E. coli* O157:H7), *Escherichia coli* is a very common inhabitant of the lower intestine and even a probiotic strain is known: *Escherichia coli* Nissle 1917 (EcN). As mentioned before *Escherichia coli* Nissle 1917 together with other probiotics have been proven to treat constipation [43] and inflammatory bowel disease [83]. This strain could also relieve gastrointestinal disorder, ulcerative colitis, Crohn’s disease [84], even colon cancer [83], however more research is necessary.

### 4.8. Claimed Health Benefits of Individual Probiotic Microorganisms

Many publications using well-designed and well-conducted trials substantiate the health benefits of specific strains of probiotics on the risk reduction and management of a variety of diseases and conditions. Some of the documented health claims of probiotics proposed by their authors include: stimulation of various components of the immune system, gut immune response and intestinal homeostasis [85]; prevention and treatment of diarrhoea [33,39,42,86]; improvement of faecal properties and microbiota, treatment of irritable bowel syndrome, inflammatory bowel disease and constipation [43,59,60,61,85,86]; prevention and treatment of *Clostridium difficile*-associated diarrhoea in adults and children [62,87]; alleviation of symptoms of lactose intolerance and other food allergies [86]; prevention of necrotising enterocolitis in preterm infants [88,89,90]; decrease in plasma cholesterol level [35,85,91]; improvement of *Helicobacter pylori* eradication regimens [92]; therapeutic effects by supporting the immune response of HIV-infected children and adults [93,94], anti-proliferative activity on tumour cells [95,96]; reduction of viral-associated pulmonary damage through controlling immune-coagulative responses and clearing respiratory viruses [37]; immune-stimulatory properties of low molecular mass molecules produced by probiotic bacteria [97]. Although probiotics have even been proposed as treatment for eczema [98], randomized controlled trials to date do not have sufficient evidence to recommend probiotics as primary prevention [99,100]. It is important to add that much more must be done to identify scientifically proven mechanisms of action with translational pre-clinical safety studies as well as rigorous clinical trials. To prove a health claim sample sizes of trials must be sufficient, heterogeneity of study designs (targeted population) must be enabled and accurate identification of strains should be conducted.

Also, several animal studies have observed positive effects of probiotic administration [101,102,103,104] which can have a consequential positive effect on public health of humans by lowering antibiotic consumption for animals used in food production. This will consequently lower the presence of drugs and multi-resistant organisms in the environment (including drinking water). Studies on animals are also the basis for *in vivo* human trials resulting in complex knowledge. However, caution should be applied, as successful use of probiotics in animal studies does not necessarily mean that similar protocols will be successful for human trials, especially for patients. Such, was the case in the study on the use of probiotics in patients suffering severe acute pancreatitis where a significantly higher mortality was observed for the probiotics group than for the placebo group [105], although these results were unexpected in the light of the results of animal studies [106]. However, this case is not representative as normally a probiotic would not be administered to this category of patients with multi-organ failure. It was later postulated that an error in the treatment, which led to the adverse cascade of events that caused organ failure and ultimately death, was perhaps due to the high incidence of gut ischemia in the treatment group [107]. 

The most common probiotic microorganisms with claimed health benefits for humans from the most recent scientific literature are noted in Table 1. Where data was available, strain specific data was added in brackets before the reference.

**Table 1:** Recently published claimed health benefits of probiotic microorganisms.

Genus	Species	Recently published health claims with references (strain specific date is noted where available)
*Lactobacillus*	*L. rhamnosus*	Reduction of viral-associated pulmonary damage (*L. rhamnosus* CRL1505) [37]; prevention and reduction of severity of atopic dermatitis in children (*L. rhamnosus* GG) [108]; reduction of risk for developing allergic disease (*L. rhamnosus* GG) [109], (*L. rhamnosus* HN001 [110]; anti-diabetic potential (various strains from human infant faecal samples) [111]; prevention of necrotizing enterocolitis in newborns (*L. rhamnosus* GG) [112]; prevention or treatment of bacterial vaginosis (*L. rhamnosus* GR-1) [113]; aid in weight loss of obese women (*L. rhamnosus* CGMCC1.3724) [114]; treatment of acute gastroenteritis in children (*L. rhamnosus* GG) [115]; reduction of risk for rhinovirus infections in preterm infants (*L. rhamnosus* GG and *L. rhamnosus* ATCC 53103) [116]; protection of human colonic muscle from lipopolysaccharide-induced damage (*L. rhamnosus* GG) [117]
	*L. acidophilus*	Treatment of travellers’ diarrhoea [39]; reduction of hospital stay of children with acute diarrhoea [118]; antifungal activity (*L. acidophilus* ATCC-4495) [119]; prevention or treatment of bacterial vaginosis [113]; treatment of *C. difficile*-associated diarrhoea [119]; reduction of incidence of febrile urinary tract infections in children [120]; reduction of irritable bowel syndrome symptoms [121].
	*L. plantarum*	Prevention of endotoxin production [35]; antifungal activity *(L. plantarum* NRRL B-4496) [119] reduction of irritable bowel syndrome symptoms [121].
	*L. casei*	Treatment of functional constipation in adults (*L. casei* Lcr35 and *L. casei* Shirota) [43]; treatment of *C. difficile*-associated diarrhoea [122]; restoration of vaginal flora of patient with bacterial vaginosis (*L. casei* Lcr35) [123]; reduction of irritable bowel syndrome symptoms [121]; reduction of diarrhoea duration of antibiotic-associated diarrhoea in geriatric patients (*L. casei* Shirota) [124]; immunomodulatory mechanisms (*L. casei* Shirota) [125]; improvement of rheumatoid arthritis status (*L. casei* 01) [126]; protection against *Salmonella* infection (*L. casei* CRL-431) [127]; prevention of *Salmonella*-induced synovitis [128]; treatment of intravaginal staphylococcosis (*L. casei* IMV B-7280) [129].
	*L. delbrueckii* subsp. *bulgaricus*	Antibiotic resistance of yogurt starter culture [130]; enhancement of systemic immunity in elderly (*L. delbrueckii* subsp. *bulgaricus* 8481) [131]; antibacterial action against *E. coli* [132]; modulation of brain activity [133].
	*L. brevis*	Protective role in bile salt tolerance (*L. brevis* KB290) [134]; reduction in plague acidogenicity (*L. brevis* CD2) [135].
	*L. johnsonii*	Impact on adaptive immunity for protection against respiratory insults [136]; reduction of occurrence of gastritis and risk of *H. pylori* infection (*L. johnsonii* MH-68) [137]; inhibition of *S. sonnei* activity (*L. johnsonii* F0421) [138]; treatment of perennial allergic rhinitis in children together with levocetirizine (*L. johnsonii* EM1) [139].
	*L. fermentum*	Prevention or treatment of bacterial vaginosis (*L. fermentum* RC-14) [113]; blockage of adherence of pathogenic microorganisms on vaginal epithelium [140]; antistaphylococcal action (*L. fermentum* ATCC 11739) [141]; potential for reduction of insulin resistance and hypercholesterolemia (*L. fermentum* NCIMB 5221) [142].
	*L. reuteri*	Reduction of low-density lipoprotein cholesterol (*L. reuteri* NCIMB 30242) [71]; treatment of acute gastroenteritis in children [115]; reduction of diarrhoea duration in children (*L. reuteri* ATCC 55730) [143]; management of infant colic (*L. reuteri* ATCC 55730 and *L. reuteri* DSM 17938) [144]; reduction of onset of gastrointestinal disorders in infants (*L. reuteri* DSM 17938) [145]; reduction of frequency of proven sepsis, feeding intolerance and duration of hospital stay in preterm infants (*L. reuteri* DSM 17938) [146].
*Bifidobacterium*	*B. infantis*	Reduction of irritable bowel syndrome symptoms [122]; reduction of necrotizing enterocolitis in preterm infants [147,148,149].
	*B. animalis* subsp. *lactis*	Treatment of functional constipation in adults (*B. animalis* subsp. *lactis* DN-173 010) [43]; reduction of incidence of febrile urinary tract infections in children [121]; modulation of brain activity [133]; reduction of necrotizing enterocolitis in preterm infants [147]; reduction of total microbial counts in dental plaque (*B. animalis* subsp. *lactis* DN-173 010) [150]; reduction of total cholesterol (*B. animalis* subsp. *lactis* MB 202/DSMZ 23733) [151]; reduction of risk of upper respiratory illness (*B. animalis* subsp. *lactis* BI-04) [152].
	*B. bifidum*	Reduction of hospital stay of children with acute diarrhoea [118]; reduction of necrotizing enterocolitis in preterm infants [148,149]; reduction of total cholesterol (*B. bifidum* MB 109/DSMZ 23731) [151].
	*B. longum*	Prevention and treatment of necrotizing enterocolitis in newborns [51]; reduction of radiation induced diarrhoea [52]; reduction of necrotizing enterocolitis with Bifidobacteria cocktail (*B. breve*, *B. infantis*, *B. bifidum*, *B. longum*) [149]; reduction of irritable bowel syndrome symptoms [122]; treatment of gastrointestinal diseases (*B. longum* CMCC P0001) [153]; perinatal intervention against onset of allergic sensitization (*B. longum* CCM 7952) [154].
	*B. breve*	Prevention and treatment of necrotizing enterocolitis in newborns [51]; reduction of necrotizing enterocolitis with Bifidobacteria cocktail (*B. breve*, *B. infantis*, *B. bifidum*, *B. longum*) [149]; reduction of cholesterol (*B. breve* MB 113/DSMZ 23732) [151].
*Saccharomyces*	*S. boulardi*	Treatment of travellers’ diarrhoea [39]; treatment and reduction of diarrhoea duration regardless of cause [7,33,56,57,58]; treatment of irritable bowel syndrome [59]; treatment of moderate ulcerative colitis [60,61]; treatment and reduction of recurrent pseudomembrane colitis infection caused by *C. difficile* [62]; treatment of acute gastroenteritis in children [115].
*Lactococcus*	*L. lactis* subsp. *lactis*	Treatment of antibiotic-associated diarrhoea [33]; adhesion to vaginal epithelial cells (*L. lactis* subsp. *lactis* KLDS4.0325) [65]; nisin production (*L. lactis* subsp. *lactis* CV56) [66]; modulation of brain activity [133]; antimicrobial activity against *C. difficile* [155]; antimicrobial and probiotic properties (*L. lactis* subsp. *lactis* ATCC 11454) [156].
*Enterococcus*	*E. durans*	Antibiotic and antioxidant activity (*E. durans* LAB18s) [70], adherence to colonic tissue and anti-inflammatory activity [157].
	*E. faecium*	Treatment of antibiotic-associated diarrhoea [34]; efficient animal probiotic [73].
*Streptococcus*	*S. thermophilus*	Reduction of irritable bowel syndrome symptoms [122]; antibiotic resistance of yogurt starter culture [130]; reduction of necrotizing enterocolitis in preterm infants [147,148].
*Pediococcus*	*P. acidilactici*	Pediocin production with antimicrobial and probiotic properties (*P. acidilactici* UL5) [156]; bacteriocin production [158]; elimination of *H. pylori* infections (*P. acidilactici* BA28) [159].
*Leuconostoc*	*L. mesenteroides*	Leucoin production, probiotic profile (survival at low pH, in presence of bile salts, in presence of pepsin) (*L. mesenteroides* B7) [160].
*Bacillus*	*B. coagulans*	Treatment of antibiotic-associated diarrhoea [34,42], treatment of bacterial vaginosis (*B. coagulans* ATCC PTA-11748) [161]; immunological support (*B. coagulans* GandenBC30) [162]; prevention of caries in children [163].
	*B. subtilis*	Efficient animal probiotic [74,75]; treatment of diarrhoea and aiding in *H. pylori* eradication (*B. subtilis* R0179) [76]; production of nitric oxide [164].
	*B. cereus*	Efficient animal probiotic (*B. cereus* NVH75/95) [80].
*Escherichia*	*E. coli* Nissle 1917	Treatment of functional constipation in adults [43]; treatment of inflammatory bowel disease [83]; treatment of gastrointestinal disorders [84]; pro-inflammatory potential [165]; prevention of surface ocular diseases [166]; reduction of *Salmonella enterica* Typhimurium intestinal colonization by iron competition [167].

Some of the health claims of individual probiotic microorganisms noted in Table 1 are based on *in vitro* using cell cultures or animal models and others are based on *in vivo* studies. From the table we can see that health claims are very diverse and range from managing various gastrointestinal diseases or disorders to exhibiting antibiotic properties and reducing total cholesterol. Some authors even claim that probiotics reduce tumour growth, modulate brain activities and reduce allergies. However, many authors stress in their conclusions that more randomized double-blinded trials need to be conducted in the future before making recommendations.

## 5. Conclusions

This article shows that the research and subsequent knowledge on microorganisms with probiotic properties is by far not complete and we are probably only reaching the tip of the iceberg as there are many different strains of microorganisms with diverse health benefits.

The literature overview shows that, although the vast majority of probiotics are generally regarded as safe (GRAS) and beneficial for healthy individuals, caution is needed in selecting and monitoring of probiotics when administering probiotics to patients with compromised immune systems, leaky gut or critical illnesses. The most common observed adverse effects include: sepsis, fungemia and gastrointestinal ischemia. Therefore, while the overwhelming existing evidence suggests that probiotics are safe, complete consideration of risk-benefit ratio before prescribing is recommended [107].

Although it is a scientific fact that certain microorganisms can kill us, the questions arise: do certain microorganisms also keep us alive? Would it be more correct to imply that the post-modern rise of conditions and diseases such as obesity, celiac disease, asthma, allergy syndromes, type 1 diabetes and Chrohn’s disease; are not only due to our genome but are also a result of the adaption of gastro-intestinal microorganisms caused by lifestyle changes, excessive intake of processed foods and sugar [7,10,168]? We cannot overlook the fact that microorganisms outnumber human cells by tenfold; the majority of which are found in the human intestinal tract [3]. Microorganisms play an important role in our gut associated lymphoid tissue, thus having profound influence on our immune system [169] and beyond. A dramatic manipulation of the intestinal microbiota involving faecal microbiota transplantation has shown remarkable clinical effectiveness for recurrent *Clostridium difficile* infection and ongoing studies are investigating effectiveness for other diseases [170], thus again proving the complexity of our intestinal microbiota.

Although the discovery of antibiotics was an important breakthrough in the 20th century and drugs such as penicillin and streptomycin have saved millions of lives, there is always collateral damage as our commensal microbiota is also affected [10,171]. Even though the intestinal microbiota is usually not permanently modulated with probiotic administration, this does not mean that during acute disruption of the sensitive intestinal microbiota balance, transiently present probiotics do not aid the permanent microbiota in restoring this balance.

With regard to probiotics it is also important to be careful with the science and not to oversell it [168,172]. Most probiotic products at the moment do not to go through pre-market approvals and are commonly used for a much wider range of scenarios in which their efficacy is not well established [173]. Therefore, future health claims concerning probiotics and their safety will critically depend on scientific evidence through science-based clinical studies on targeted population [174]. Another important issue is correct strain identification of each probiotic, for which expanding the internationally recognised culture collections of taxonomically classified and deposited probiotics is necessary [172]. This would assure strict use only of tested strains [175] with known profiles in compliance with regulatory pathways for ‘fit for human use consumption’ protocols.

Perhaps it will be possible that in the future probiotics will be used as approved drugs that will be prescribed together with/or instead of antibiotics for certain conditions such as ear infections or sinusitis [10]? An interesting concluding thought of this overview is that even though it is true that certain microorganisms can make us sick and even kill us, certain microorganisms are beneficial.
